# Bacteriophage and Phage-Encoded Depolymerase Exhibit Antibacterial Activity Against K9-Type *Acinetobacter baumannii* in Mouse Sepsis and Burn Skin Infection Models

**DOI:** 10.3390/v17010070

**Published:** 2025-01-06

**Authors:** Alexander I. Borzilov, Nikolay V. Volozhantsev, Olga V. Korobova, Lyubov V. Kolupaeva, Evgenia S. Pereskokova, Tatiana I. Kombarova, Mikhail M. Shneider, Konstantin A. Miroshnikov, Ivan A. Dyatlov, Anastasia V. Popova

**Affiliations:** 1 State Research Center for Applied Microbiology and Biotechnology, City District Serpukhov, Moscow Region, 142279 Obolensk, Russia; borzilov@obolensk.org (A.I.B.); nikvol@obolensk.org (N.V.V.); korobova@obolensk.org (O.V.K.); melstryder@yandex.ru (L.V.K.); pereskokova@obolensk.org (E.S.P.); kombarova@obolensk.org (T.I.K.); dyatlov@obolensk.org (I.A.D.); 2 Shemyakin-Ovchinnikov Institute of Bioorganic Chemistry, Russian Academy of Sciences, 117997 Moscow, Russia; mikhailshneider@gmail.com (M.M.S.); kmi@bk.ru (K.A.M.)

**Keywords:** *Acinetobacter baumannii*, bacteriophage, depolymerase, mouse sepsis model, mouse burn skin infection model

## Abstract

*Acinetobacter baumannii* is a widely distributed nosocomial pathogen that causes various acute and chronic infections, particularly in immunocompromised patients. In this study, the activities of the K9-specific virulent phage AM24 and phage-encoded depolymerase DepAPK09 were assessed using in vivo mouse sepsis and burn skin infection models. In the mouse sepsis model, in the case of prevention or early treatment, a single K9-specific phage or recombinant depolymerase injection was able to protect 100% of the mice after parenteral infection with a lethal dose of *A. baumannii* of the K9-type, with complete eradication of the pathogen. In the case of delayed treatment, mouse survival decreased to 70% when injected with the phage and to 40% when treated with the recombinant enzyme. In the mouse burn skin infection model, the number of *A. baumannii* cells on the surface of the wound and in the deep layers of the skin decreased by several-fold after treatment with both the K9-specific phage and the recombinant depolymerase. The phage and recombinant depolymerase were highly stable and retained activity under a wide range of temperatures and pH values. The results obtained contribute to expanding our understanding of the in vivo therapeutic potential of specific phages and phage-derived depolymerases interacting with *A. baumannii* of different capsular types.

## 1. Introduction

*Acinetobacter baumannii* is a Gram-negative, strictly aerobic, non-fermentative, oxidase-negative, catalase-positive bacterium that causes a range of healthcare-associated infections, including ventilator-associated pneumonia and bloodstream, urinary tract, central nervous system, skin, and soft tissue infections, especially in intensive care and burn units [1,2,3,4]. *A. baumannii* is a representative of the ESKAPE group (*Enterococcus faecium*, *Staphylococcus aureus*, *Klebsiella pneumoniae*, *Acinetobacter baumannii*, *Pseudomonas aeruginosa*, and *Enterobacter* spp.), the members of which are characterized by resistance to multiple classes of antibiotics and are prevalent as causative agents of nosocomial infections throughout the world [5,6,7]. Most *A. baumannii* isolates are multidrug-resistant (non-susceptible to at least one agent in three or more antimicrobial classes), extensively drug-resistant (non-susceptible to at least one antibiotic agent in all but two or fewer antimicrobial classes), or pan drug-resistant (non-susceptible to all agents in all standard-of-care antimicrobial categories), which is a serious challenge in clinical practice [5,8] *A. baumannii* strains carrying metallo-*β*-lactamases and oxacillinase serine *β*-lactamases show resistance to carbapenems, which, along with colistin, are the last resort antibiotics for combating this pathogen [5,9,10]. Therefore, carbapenem-resistant *A. baumannii* was included as a critical priority microorganism by the World Health Organization (WHO) and represents one of the biggest threats to public health [11].

The global problem associated with the spread of multiresistant pathogens has necessitated the development of new antimicrobial drugs. In the search for alternative or complementary therapeutics, increasing attention is being paid to bacteriophages and phage-encoded enzymes. Many phages contain tail-associated structural proteins with polysaccharide-degrading activity or tailspike depolymerases [12,13]. These enzymes are involved in the destruction of surface bacterial structures, such as capsular polysaccharides, exopolysaccharides, or carbohydrate components of lipopolysaccharides. In the case of *A. baumannii* and other clinically relevant representatives of the species *Acinetobacter*, the capsular polysaccharides (CPSs) surrounding the bacterial cells are the primary receptors for the phages bearing tailspike proteins participating in the initial step of phage–host interaction [14,15,16]. More than 240 different capsule biosynthesis gene clusters (KL) have been bioinformatically predicted in *Acinetobacter* spp. genome assemblies deposited in the National Center for Biotechnology Information (NCBI) database [17], implying the existence of at least an equal number of phage-encoded CPS-degrading enzymes. Thus, the structures of depolymerases determine the specificity of phages to particular capsule types of *A. baumannii* [18].

CPSs are important factors for the survival and virulence of bacterial cells [19,20], and capsule-specific phages and phage-derived depolymerases can act as potential “antivirulence” agents. In recent years, the antivirulent efficacy of several *A. baumannii* phages and phage-encoded depolymerases has been explored in different mouse models [21,22,23,24,25,26,27,28]. In this study, the previously comprehensively characterized K9-specific virulent phage AM24 [29] and phage-derived depolymerase DepAPK09 [16] were investigated for their applications in controlling *A. baumannii* infections in vivo in mouse sepsis and burn skin infection models. Moreover, the stabilities of the phage and the depolymerase under different conditions were tested. Considering that both phage AM24 and APK09 phage-derived depolymerase interact specifically with extensively drug-resistant *A. baumannii* strain B05, which was assigned to sequence type 2 according to the Pasteur multilocus sequence typing scheme [30], the results obtained allow us to evaluate the prospects of their possible usage in combating a significant nosocomial pathogen belonging to the prevalent worldwide international clone IC2.

## 2. Materials and Methods

### 2.1. Bacterial and Bacteriophage Growth Conditions

Phage AM24 and its bacterial host strain, *A. baumannii* B05 (GenBank accession number: JAROBQ000000000), were obtained from the State Collection of Pathogenic Microorganisms and Cell Cultures «SCPM-Obolensk» (accession numbers SCPM-O-Ph-106 and SCPM-O-B-8162, respectively). Phage AM24 was originally isolated from clinical materials obtained from the N.N. Burdenko Research Institute for Neurosurgery (Moscow, Russia) in 2014 [29].

Bacteria were cultured in Luria–Bertani (LB) broth (Difco Laboratories, Detroit, MI, USA) or on LB broth agar plates at 37 °C.

### 2.2. Preparation of Phage AM24 and the Recombinant Protein DepAPK09 for In Vivo Experiments

The phage AM24 was propagated using a liquid culture of *A. baumannii* B05 (OD_600_ of 0.3) at a multiplicity of infection (MOI) of 0.1. Phage particles were purified using the PEG/NaCl method [31] and dissolved in saline solution (0.9% sodium chloride solution).

The expression vector pTSL [32], which contains the gene fragment corresponding to DepAPK09 lacking the N-terminal domain [16], was transformed into chemically competent *Escherichia coli* B834 (DE3) cells. Protein expression was performed in 2xTY medium (16 g of tryptone, 10 g of yeast extract, 5 g of NaCl) supplemented with ampicillin at 100 µg/mL. The transformed cells were grown at 37 °C until the optical density reached 0.6 at 600 nm. The medium was cooled to the temperature of 18 °C, followed by expression induction by the addition of isopropyl-1-thio-β-d-galactopyranoside (IPTG) to a final concentration of 1.0 mM. After incubation for 16 h at 18 °C, the cells were harvested by centrifugation at 3500× *g* for 20 min at 4 °C. The cell pellets were then resuspended in buffer A (20 mM Tris pH 8.0, 0.3 M NaCl) and sonicated (Virsonic, VirTis, France). The lysates were cleared via centrifugation at 15,000× *g* for 20 min and then loaded into 5-mL Ni^2+^-charged GE HisTrap columns (GE Healthcare Life Sciences, Chicago, IL, USA) equilibrated with buffer A. The proteins were eluted using a 50–200 mM imidazole step gradient in buffer A. His-tag and SlyD digestion was achieved by incubation with tobacco etch virus (TEV) protease at a protease/protein ratio of 1/100 (wt/wt) overnight with simultaneous dialysis against 10 mM Tris pH 8.0 containing 1.0 mM 2-mercaptoethanol. The cleaved protein was loaded onto a 5 mL SourceQ 15 (GE Healthcare Life Sciences, Chicago, IL, USA) column and eluted with a linear gradient of 0–1 M NaCl in 20 mM Tris-HCl (pH 8.0). Protein-containing fractions were combined and concentrated to ~10 mg/mL using Sartorius ultrafiltration devices with a molecular mass weight cutoff of 50 kDa (Sartorius AG, Gottingen, Germany). The protein concentration was determined using the Bradford method with bovine serum albumin (BSA) as the standard.

### 2.3. Phage AM24 Infection Inhibition Assay

The AM24–DepAPK09 infection inhibition assay was performed according to a published procedure [33]. A titer of 2.0 × 10^7^ PFU/mL for the phage AM24 was chosen for the competition experiments. *A. baumannii* B05 was grown in LB medium at 37 °C to an OD_600_ of 0.3. DepAPK09 was then added to a 100-μL aliquot of the cell culture to a final concentration of 0.5 mg/mL and incubated for 25 min at 37 °C. One hundred-microliter aliquots of *A. baumannii* B05 host cells with no additives and a final BSA concentration of 0.5 mg/mL incubated for 25 min at 37 °C served as controls. After incubation, several 10-fold dilutions of phage AM24 and 4 mL of soft agar (LB broth supplemented with 0.6% agarose) were added to the mixtures and plated onto the nutrient agar. The plates were incubated overnight at 37 °C, and the number of AM24 plaques was determined. The experiment was performed in triplicate.

### 2.4. Phage AM24 and Recombinant Depolymerase Stability

To estimate AM24 pH-dependent stability, the phage was added to SM buffer (10 mM Tris-HCl, 10 mM MgSO_4_, and 100 mM NaCl), with adjusted pH values of 3.0, 5.0, 7.0, 9.0, and 11.0 using 5 M HCl or 5 M NaOH, to final concentration of 1 × 10^7^ PFU/mL and incubated for 1 h at room temperature. To estimate DepAPK09 pH-dependent stability, the depolymerase was added to different buffer systems (50 mM sodium acetate buffer, 50 mM sodium phosphate buffer, 50 mM Tris-HCl buffer) with adjusted pH values of 3.0, 5.0, 7.0, 9.0, and 11.0 to final concentration of 100 µg/mL and incubated for 1 h at room temperature. To assess thermostability, the preparations were incubated in SM buffer at temperatures ranging from 4 °C to 92 °C for 1 h. The efficiency of plating (EOP) of phage AM24 after different treatments was determined using the double-layer agar method [34]. To determine depolymerase activity, serial twofold dilution drops corresponding to a 1–0.0078 μg range were spotted onto agar plates overlaid with a mixture of *A. baumannii* B05 bacterial culture with 4 mL of soft agar, and the maximal dilution that formed a translucent halo on the bacterial lawn was determined. All experiments were performed in triplicate.

### 2.5. In Vivo A. baumannii Infection Models

Two mouse models of *A. baumannii* primary sepsis and burn infection were used to evaluate the therapeutic efficacy of phage AM24 and recombinant depolymerase DepAPK09. White BALB/c mice (females; age, 9 to 10 weeks; weight, 18 to 20 g; Stolbovaya laboratory animal nursery, Moscow Region, Russia) and white outbred mice (females/males; age, 8 to 10 weeks; weight, 24 to 27 g; Stolbovaya laboratory animal nursery, Moscow Region, Russia) were used in the study. Mice were housed in polycarbonate cages (Lab Products Inc., Seaford, DE, USA), with five animals in each; they had access to food and water ad libitum and were subjected to daily veterinary observation. Mice that died during the experiment were immediately removed from the cages. All animal procedures were approved by the BioEthic Committee of the State Research Center for Applied Microbiology and Biotechnology and met the guidelines of Directive 2010/63/EU of the European Parliament and of the Council of 22 September 2010 on the protection of animals used for scientific purposes.

In the sepsis model, BALB/c mice of the control and six experimental groups (10 mice in each) were infected intraperitoneally with a culture of *A. baumannii* B05 at a dose of 6.3 × 10^5^ CFU, equivalent to 40 LD_50_, in a saline solution containing 2.5% mucin from porcine stomach (Sigma-Aldrich, Burlington, MA, USA). Depolymerase DepAPK09 was injected into mice in three experimental groups (once, intraperitoneal, 50 µg per mouse) 1 h before infection (hbi), half an hour past infection (hpi), and 6 hpi. The phage AM24 at a dose of 10^9^ PFU was administered to mice according to the same regimen: once intraperitoneally to mice in three groups at 1 hbi, 1.5 hpi, and 6 hpi. The control group did not receive the phage or depolymerase. All animals were sacrificed 10 days after infection, and blood and parenchymal organs (lungs and liver) were examined for the presence of *A. baumannii* cells. Mice that died during the experiment were also autopsied and examined for bacterial dissemination. The effectiveness of the drugs was assessed according to the degree of protection against the death of mice and *A. baumannii* B05 contamination of their internal organs and blood.

In the burn skin infection model, 15 white outbred mice were randomly distributed into three groups of five mice each. The burn procedure was performed under general anesthesia via subcutaneous injection of a zoletil–xylazine cocktail. A skin fold was formed on a previously shaved area of skin using two tweezers. For 60 s, the fold was clamped on both sides with two metal plates measuring 1 × 1 cm that were preheated in boiling water for 5 min. As a result, a burn surface with an area of 2 cm^2^ was formed on the skin. Immediately after the formation of the burns, mice were intraperitoneally resuscitated with 0.5 mL of sterile saline to prevent dehydration. After 5 min, the wound was infected with a culture of *A. baumannii* B05, distributing 40 μL of a bacterial suspension with a concentration of 2.5 × 10^9^ CFU/mL over the burn surface. The infected wound was covered with a napkin gauze swab moistened with saline and fixed with an elastic bandage around the body (Figure S1). The bandages were removed 24 h after infection, and 30 μL of a preparation containing either 1 × 10^9^ PFU of phage AM24, 50 μg of depolymerase DepAPK09, or saline (control group of mice) was applied to the wounds. The treatment was repeated twice with an interval of 6 h. In this case, napkins and bandages were not used. All mice were euthanized 24 h after treatment, and the concentration of *A. baumannii* was determined in the skin and washed off the burn wound surface. Drug effectiveness was assessed according to the level of *A. baumannii* contamination of the wound surface and deep skin layers in the control and experimental groups.

### 2.6. Statistical Analysis

The data computation and statistical tests were performed using GraphPad Prism 8.0 software (GraphPad Software, Inc., La Jolla, CA, USA). Statistical significance was determined using a *t*-test. *p* < 0.05 was considered statistically significant.

## 3. Results

### 3.1. Overall Characteristics of the K9-Specific Phage AM24 and Depolymerase DepAPK09 Used in In Vivo Experiments

Phage APK09, from which the specific depolymerase DepAPK09 was derived, and phage AM24 were isolated and were able to infect the same bacterial host, *A. baumannii* B05, belonging to the K9 capsular type [16,29]. This indicates that the phages carry tailspike depolymerases, which specifically recognize and degrade the CPS of the same K9 structure. The depolymerases encoded in the genomes of the phages APK09 and AM24 do not share a high level of amino acid similarity with each other (the coverage obtained to an E-value of 3 × 10^−48^ was 76% with an identity 28.57%). However, the structural similarity of the CPS-recognizing/degrading parts of the experimentally determined monomers of DepAPK09 (GenBank accession number: UAW09804; PDB ID: 8OQ0) and AM24_gp50 (GenBank accession number: APD20249; PDB ID: 5W5P) was very high [16].

**Figure 1:**
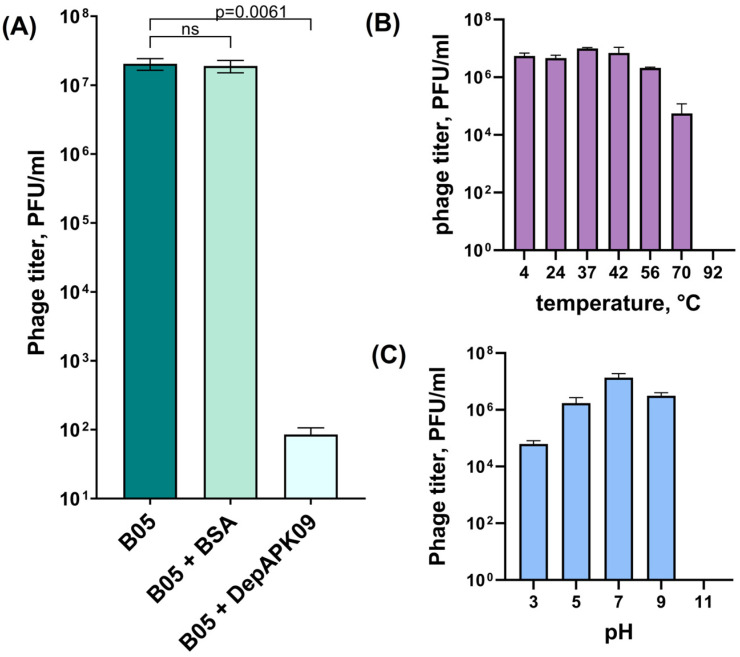
(**A**) Phage AM24 infection inhibition by DepAPK09. From left to right, phage titers were observed on the bacterial lawns after the treatment of *A. baumannii* B05 cells with phage AM24 only, after cell cultures were preincubated with BSA (as a negative control), and with purified DepAPK09, followed by phage AM24 treatment. Significance was determined using the *t*-test. *p*: *p*-value, ns: not significant. (**B**) Activity of phage AM24 in a range of different temperatures during 1 h of incubation. (**C**) Stability of phage AM24 in various pH conditions during 1 h of incubation. The error bars represent the standard deviation of the mean phage titers.

To demonstrate that DepAPK09 and phage AM24 equally effectively interact with the same surface receptor, namely, the *A. baumannii* CPS with the K9 structure, competition experiments were performed (Figure 1A). For this purpose, *A. baumannii* B05 bacterial culture, preincubated with purified DepAPK09, was mixed with several phage AM24 dilutions and plated on agar plates. In negative-control experiments, host bacterial cells were either not pretreated or 0.5 mg/mL of BSA was added. After overnight incubation, the phage titer was measured. Pretreatment of *A. baumannii* B05 with DepAPK09 significantly inhibited AM24–host binding due to the cleavage of the K9 CPS, the primary receptor for the phage, whereas coincubation of host bacterial cells with BSA did not result in significant differences in phage titers.

The optimal activities and stabilities of phage AM24 and the recombinant depolymerase DepAPK09 with respect to pH and temperature were further determined. The results of the experiments showed that the phage retained its infectivity in a range of temperatures between 4 °C and 56 °C, with a partial loss of its ability to infect at 70 °C and a complete loss of this ability at 92 °C (Figure 1B). The optimal pH values for phage AM24 were between 5.0 and 9.0, but the phage partially lost its activity at a pH of 3.0 and completely lost its activity at a pH of 11.0 (Figure 1C). Recombinant depolymerase was thermostable and retained activity over a range of temperatures from 4 °C to 56 °C, with complete loss of activity at 70 °C. It remained active from a 0.0078–1 μg range under pH values between 5.0 and 9.0. When the protein was exposed to a pH of 3.0, it showed enzymatic activity in a 0.0315–1 μg range. Examples of DepAPK09 spot tests under different conditions are presented in Figure S2.

### 3.2. Therapeutic Potential of the Phage AM24 and DepAPK09 in Mouse Models of A. baumannii Infection

*A. baumannii* causes multiple types of infection, especially in immunocompromised patients in intensive care and burn units [1]. Therefore, the therapeutic efficacy of AM24 and DepPK09 preparations was evaluated in laboratory models that differed in the route of infection and infectious process development, namely, the mouse models of primary sepsis and burn infection.

**Figure 2:**
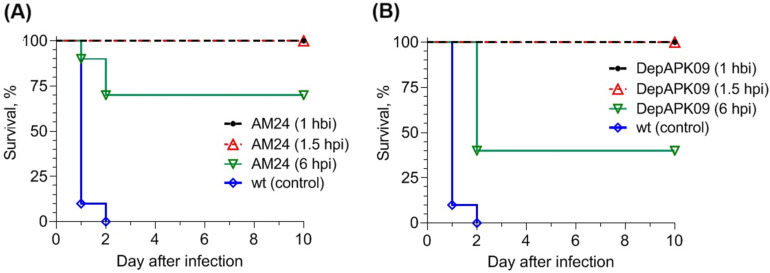
Protective effects of phage AM24 (**A**) and depolymerase DepAPK09 (**B**) in mice intraperitoneally challenged with *A. baumannii* B05 (6.3 × 10^5^ CFU) in a solution containing 2.5% mucin. Phage (10^9^ PFU per mouse) or depolymerase (50 μg per mouse) was administered once intraperitoneally 1 h before infection (hbi), 1.5 h after infection (hpi), or 6 h after infection with *A. baumannii* B05. WT: without treatment. Uninfected mice receiving the phage or depolymerase only were used as experimental controls. The survival rate of these groups was 100%; thus, for simplicity, these groups are not included in the figure.

To model mouse sepsis, the clinical strain *A. baumannii* B05, which causes 100% death in BALB/c mice intraperitoneally administered at a dose of 40 LD_50_, was used in a solution with 2.5% mucin. Phage and depolymerase were administered to animals according to the same regimen: once intraperitoneally at a dose of 10^9^ PFU (plaque-forming units) and 50 μg/mouse, respectively, 1 h before infection (prevention), 1.5 h after infection (early treatment), and 6 h after infection (delayed treatment). The results of phage AM24 and depolymerase DepAPK09 treatment in mice showed that the best therapeutic effect was achieved with prevention and early treatment. In both cases, all mice were alive 10 days after the end of therapy, whereas all mice in the control group without treatment died 2 days after infection. With delayed treatment, 6 h after infection, mouse survival decreased to 70% when treated with phage AM24 and to 40% when treated with depolymerase DepAPK09 (Figure 2A,B). The surviving mice did not exhibit either general or local signs of infection 10 days after treatment. Bacteriological analysis of the organs and tissues of these animals revealed that they did not contain *A. baumannii* cells.

**Figure 3:**
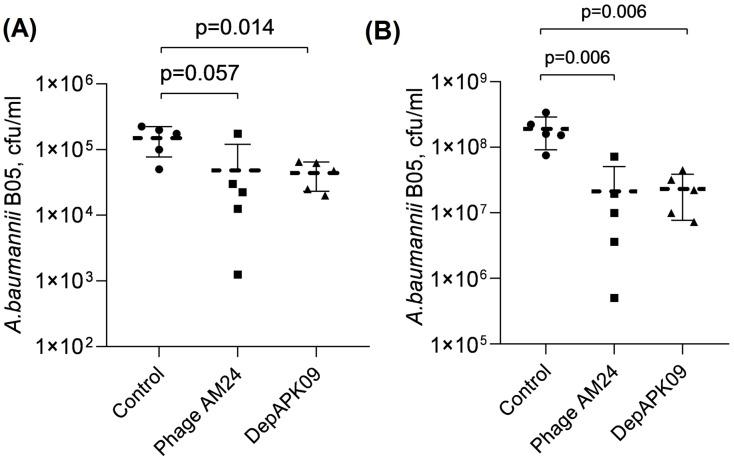
In vivo activity of phage AM24 and recombinant depolymerase DepAPK09 in a mouse burn skin infection model of *A. baumannii*. (**A**) The bacterial burden on the thermal wound surface 48 h after challenge. (**B**) The bacterial burden in the deep layers of the mouse skin 48 h after challenge. Dashed horizontal lines indicate the geometric mean values of each group; error bars represent geometric standard deviations. Significance was determined using the *t*-test. *p*: *p*-value, Control: group of mice treated with saline; Phage AM24: group of mice treated with 10^9^ PFU of phage AM24; DepAPK09: group of mice treated with 50 µg of recombinant depolymerase DepAPK09.

*A. baumannii* is a leading cause of burn and wound infections [1]; therefore, studies assessing the therapeutic efficacy of phage AM24 and recombinant depolymerase DepAPK09 in a burn skin infection model are essential. Mice thermal wounds were treated with a dose of 10^9^ PFU of phage AM24 or 50 µg of recombinant depolymerase DepAPK09 24 h after challenge with *A. baumannii* B05. The treatment was repeated twice with an interval of 6 h. The therapeutic effectiveness of the phage AM24 and depolymerase DepAPK09 was assessed by the level of colonization of the wound surface (washes from the wound surface) and deep layers of skin (skin flap homogenates) of euthanized animals 48 h after infection. Figure 3 shows the results of bacterial seeding from the wound surface (Figure 3A) and deep skin layers of mice (Figure 3B), which indicate a decrease in the level of colonization of thermal wounds with *A. baumannii* B05 24 h after treatment with both phage AM24 and depolymerase DepAPK09. The bacterial burdens on the surface of the wound and in the deep layers of the skin decreased by 3 and 10 times, respectively.

## 4. Discussion

K9-specific phages are common among all capsule-specific *Acinetobacter* bacterial viruses classified into different taxonomic groups. At least nine phages with established or predicted K9 specificity have been isolated in China, Portugal, Russia, and the United Kingdom [18], suggesting a wide spread of *A. baumannii* bacterial hosts of the K9 type around the world. This is confirmed by the fact that KL9 was found to be among the most common K loci identified in 6.57% of 8994 *A. baumannii* genome assemblies [17].

Phage AM24 and APK09-encoded depolymerase have been comprehensively characterized in previous studies [16,29]. AM24 represents the unclassified *Caudoviricetes* group, which includes phages with myovirus morphology [18,29]. Specific depolymerase DepAPK09 was derived from phage APK09, which is a member of the genus *Friunavirus* (subfamily *Beijerinckvirinae*, family *Autographiviridae*) [16], the largest group of known phages infecting *A. baumannii* [18], including phages with a typical podovirus morphology. Recombinant depolymerase DepAPK09 was shown to be a specific glycosidase that cleaves K9 CPSs via the hydrolytic mechanism with the production of dimers and trimers of the repeating K unit. The antivirulence potential and therapeutic efficacy of recombinant DepAPK09 have been already shown using a *Galleria mellonella* model. In the performed experiments, DepAPK09 significantly inhibited the *A. baumannii*-induced death of G. mellonella larvae, demonstrating its potential as a therapeutic agent against infections caused by *A. baumannii* belonging to the K9 type [16].

In this study, competition experiments were performed to demonstrate that the depolymerase DepAPK09 and phage AM24 effectively interact with the same receptor, CPS with a K9 structure, on the cell surface of *A. baumannii* B05. The stability and therapeutic efficacy of the K9-specific phage and phage-derived depolymerase in mouse sepsis and burn skin infection models were then evaluated. The data obtained demonstrate the effectiveness of phage AM24 and recombinant depolymerase DepAPK09 in the prophylactic and early treatment of lethal sepsis in mice caused by *A. baumannii* of the K9 capsular type. Depolymerase protects 100% of animals when administered once at a dose of 50 μg/mouse 1 h before infection and 1.5 h after infection with a lethal dose of the *A. baumannii* B05. The same results were observed for the phage AM24 in the case of prevention or early treatment. Delayed treatment 6 h after infection was less effective; however, the survival rates of mice were 40% and 70% for DepAPK09 and AM24, respectively, with all untreated animals dying within the first day. Thus, intraperitoneal injection of K9-specific phage and phage-derived depolymerase effectively protected mice infected with *A. baumannii* B05.

Several previous studies have also demonstrated the effectiveness of specific phages in murine sepsis or bacteremia models [21,22,23,24,27,28]. For example, simultaneous administration of different MOIs (multiplicity of infection, MOI = 10, 1, or 0.1) of phage ϕkm18p with an intraperitoneal injection of 2–3 × 10^8^ CFU of *A. baumannii* host strain protected 100% of BALB/c mice; however, the survival rate decreased to 56% when the mice received the phage 1 h after challenge [21]. In the other work, the survival rate of BALB/c sepsis mice after seven days in the case of a single intraperitoneal administration of 10^9^ PFU of phage PD6A3 1 h after infection with 10^9^ CFU of host *A. baumannii* strain was 60% [22]. A recent study also showed that after five days of treatment, the survival rate of mice intraperitoneally injected with 10^7^ CFU/mouse and then with phage Ab_WF01 at an MOI of 0.001 two hours post-infection was 60% [28].

Moreover, some studies have also shown that a single administration of specific depolymerases via intraperitoneal injection could save animals in murine models of sepsis and systemic infections, reducing the virulence of *A. baumannii* [25,26]. In one study, it was shown that the survival rate of BALB/c mice treated with depolymerase Dpo48 (50 μg) at 2 h postinfection with 10^7^ CFU of the corresponding *A. baumannii* strain was 100% within the seven-day observation period [25]. In another study, a single administration of K2-specific depolymerase (50 μg/mouse) 1 h after challenge with 10^7^ CFU of *A. baumannii*, belonging to the K2 type exerted a significant therapeutic effect by rescuing 60% of mice and reducing local inflammation [26].

The in vivo ability of phage AM24 and recombinant depolymerase DepAPK09 to reduce the bacterial burden in the wound surface and deep skin layers of mice makes them possible candidates for local application to control *A. baumannii* wound infections.

Phage AM24 was highly stable under extreme pH and temperature conditions. Depolymerase DepAPK09 also retained its activity across a pH range of 5.0–9.0 and temperatures from 4 °C to 56 °C and was inactivated only at 70 °C. These data correlate with previous results obtained for K2-specific depolymerase, where the authors noted that the loss of enzymatic activity at temperatures of ≥70 °C was related to the loss of protein structure observed during CD spectroscopy measurements [26].

The results of in vivo experiments and stability tests revealed the promising potential of the K9-specific phage AM24 and the recombinant depolymerase DepAPK09 as possible candidates for controlling nosocomial infections caused by *A. baumannii*.

## 5. Conclusions

In this study, the therapeutic and prophylactic efficacy of K9-specific phage and phage-derived depolymerase was demonstrated in mouse sepsis models. It has also been shown that the burden of *A. baumannii* cells on the surface of the wound and in the deep layers of the skin after treatment with both specific phage and recombinant depolymerase decreased by several-fold in a mouse burn skin infection model. The results obtained contribute to determining the potential for practical usage of specific phages and phage-derived depolymerases to combat *A. baumannii* of different capsular types.

## Data Availability

Data are contained within the article.

## Supplementary Materials

The following supporting information can be downloaded at: https://www.mdpi.com/article/10.3390/v17010070/s1, Figure S1: An infected burn wound in mice. (A) A burn is caused by heated metal plates. (B) The *A. baumannii* B05 culture is applied to the wound surface. (C) The wound surface is covered with a napkin moistened with saline and fixed with an elastic bandage around the body. (D) Surface of the burn wound. (E) Hematoxylin and eosin-stained sections of the burn skin at second day post-burn. The arrows designate escha (orange), distorted hair follicles (green), and edematous subcutaneous connective tissue (blue); Figure S2. Examples of DepAPK09 spot tests on the lawn of *A. baumannii* B05 culture under different pH conditions. (A) Spot test of DepAPK09 in a 0.0078–1 μg range under pH 9.0. (B) Spot test of DepAPK09 in a 0.0078–1 μg range under pH 3.0. (C) Spot test of DepAPK09 in a 0.0078–1 μg range under pH 11.0. (D) Spot test of DepAPK09 in a 0.0078–1 μg range at 4 °C. (E) Spot test of DepAPK09 in a 0.0078–1 μg range at 70 °C.
