# Supplementary material for: Bacteriophage and Phage-Encoded Depolymerase Exhibit Antibacterial Activity Against K9-Type Acinetobacter baumannii in Mouse Sepsis and Burn Skin Infection Models
Source: Viruses. 2025 Jan 6;17(1):70. doi: 10.3390/v17010070 (PMC11768871; doi:10.3390/v17010070)

Supplementary materials.

Figure S1. An infected burn wound in mice. **(A)** A burn is caused by heated metal plates. **(B)** The *A. baumannii* B05 culture is applied to the wound surface. **(C)** The wound surface is covered with a napkin moistened with saline and fixed with an elastic bandage around the body. **(D)** Surface of the burn wound. **(E)** Hematoxylin and eosin-stained sections of the burn skin at second day postburn. The arrows designate escha (orange), distorted hair follicles (green), and edematous subcutaneous connective tissue (blue)

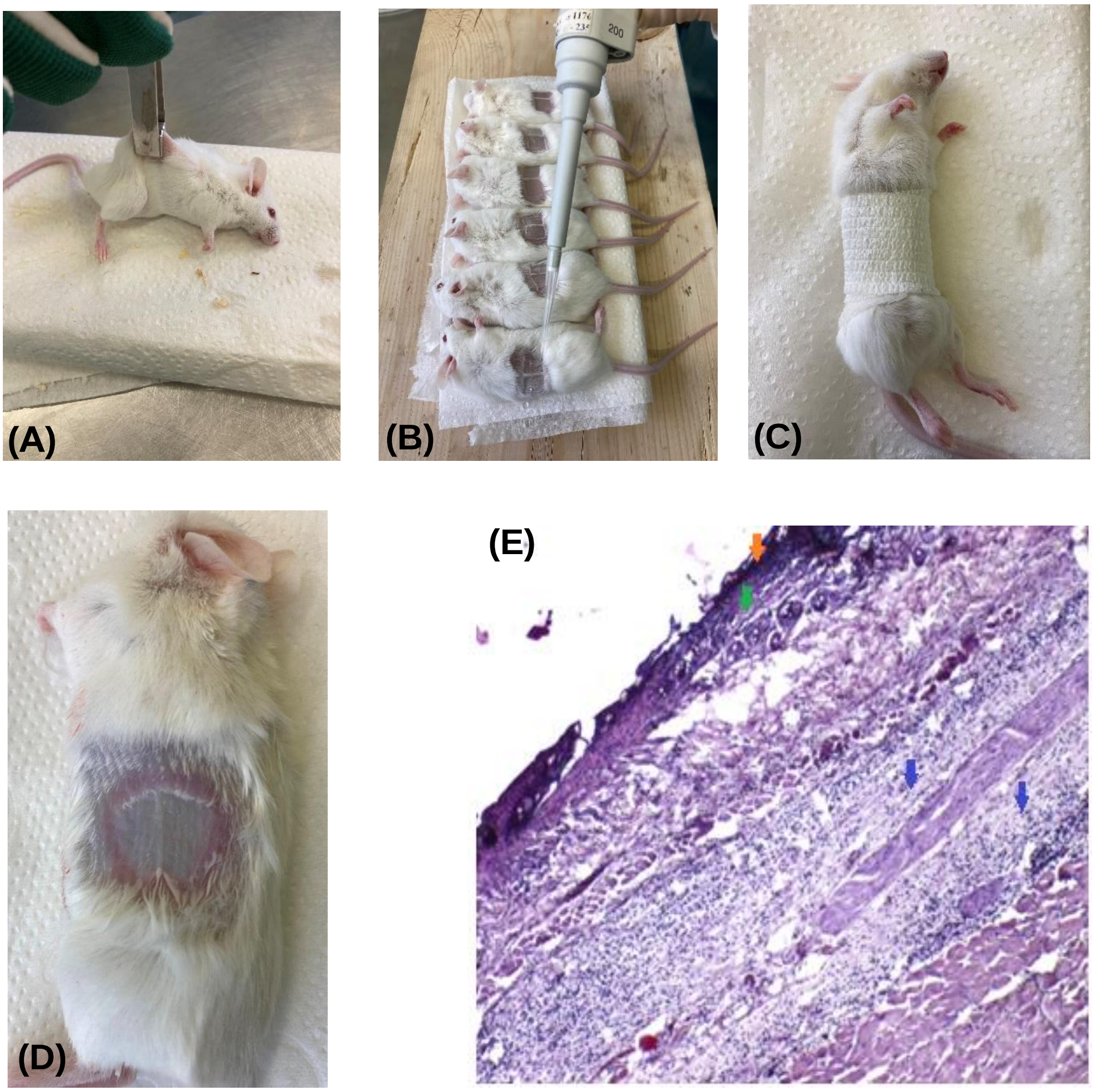

Figure S2. Examples of DepAPK09 spot tests on the lawn of *A. baumannii* B05 culture under different pH conditions. **(A)** Spot test of DepAPK09 in a 0.0078–1 μg range under pH of 9.0. **(B)** Spot test of DepAPK09 in a 0.0078–1 μg range under pH of 3.0. **(C)** Spot test of DepAPK09 in a 0.0078–1 μg range under pH of 11.0. **(D)** Spot test of DepAPK09 in a 0.0078–1 μg range at 4°C. **(E)** Spot test of DepAPK09 in a 0.0078–1 μg range at 70°C.

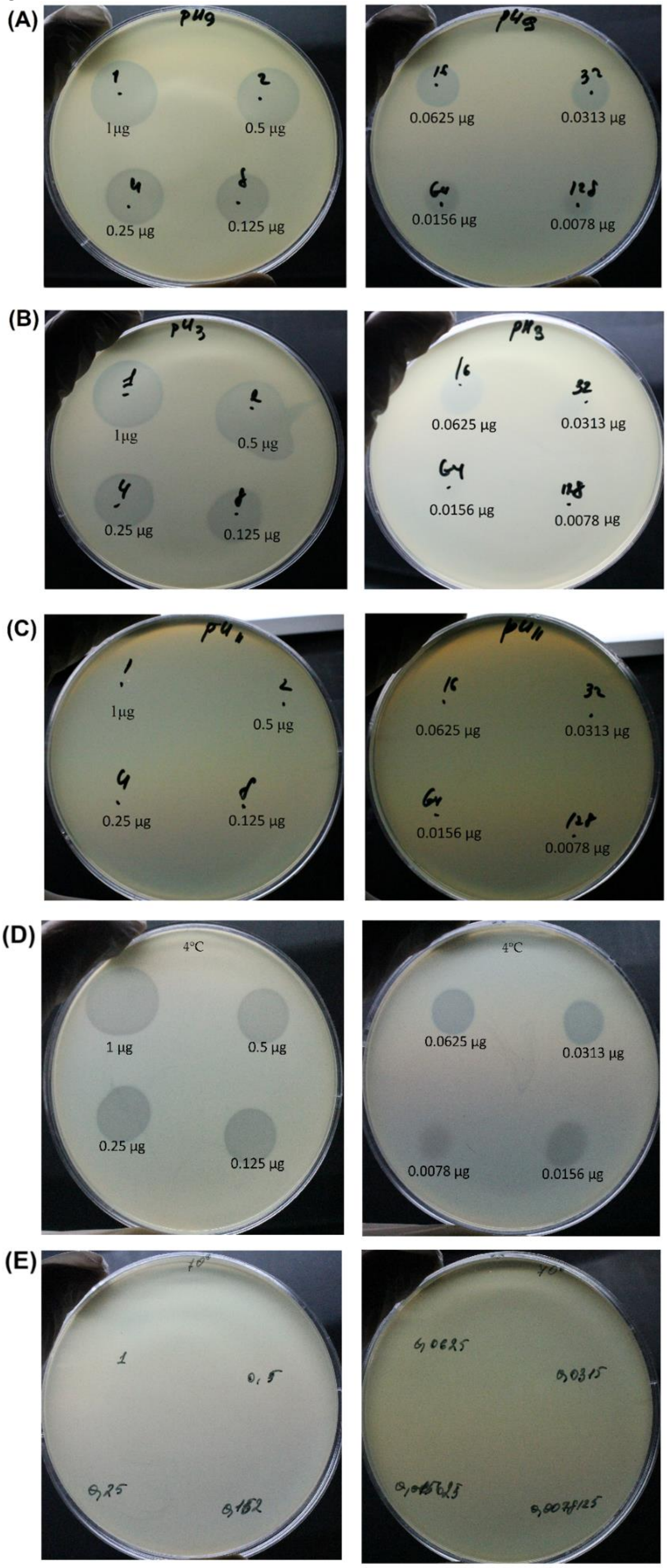

Supplement: Supplementary file 1 [file viruses-17-00070-s001.zip › viruses-3346296-supplementary.pdf]
